# Anthropogenic Disruption Versus Natural Restoration: *Enterobacter cloacae*‐Driven Barnacle Larval Settlement and Its Mitigation via Natural Bacteriophages

**DOI:** 10.1111/1751-7915.70278

**Published:** 2026-01-27

**Authors:** Atif Khan, Akash Saha, Hiren M. Joshi

**Affiliations:** ^1^ Water & Steam Chemistry Division, BARC Facilities Kalpakkam Tamil Nadu India; ^2^ Homi Bhabha National Institute Mumbai Maharashtra India

**Keywords:** bacteriophages, biofilms, biofouling, *Enterobacter cloacae*

## Abstract

Coastal regions support approximately 60% of the global population and face escalating anthropogenic pollution, which disrupts the dynamics of marine and coastal ecosystems. The discharge of over 80% of sewage without adequate treatment introduces human pathogenic microorganisms into coastal waters, posing significant risks to ecological integrity and public health. This challenge is exacerbated by cross‐resistance between antibiotics and biocides, whereby biocide use for biofilm control in coastal industries may inadvertently select for resistant pathogens of terrestrial origin. While microbial biofilms are known to promote macrofouling by facilitating invertebrate larval settlement, a major operational challenge for marine industries, the role of anthropogenic microbial contamination in influencing macrofouling dynamics remains poorly understood. Here, we provide evidence linking anthropogenic microbial contamination to marine biofouling. We isolated an antibiotic and biocide‐resistant *Enterobacter cloacae* strain from a marine cooling water circuit at an operational power plant and identified it as a potent inducer of barnacle (*Amphibalanus reticulatus*) larval settlement. Salt‐tolerance assays combined with Multi‐Locus Sequence Typing (MLST) and AAI/ANI‐based comparative genomics against reference strains indicated a likely terrestrial origin for this isolate. Larval settlement and choice assays demonstrated that *E. cloacae* biofilms increased barnacle settlement by > 70% relative to controls. To develop sustainable mitigation strategies against this biocide‐resistant organism, we isolated natural bacteriophages targeting *E. cloacae* from the same water sample. Phage‐mediated selective elimination of *E. cloacae* from biofilms reduced larval settlement by 80% in plate‐based assays, providing proof of concept for bacteriophage‐based targeted elimination of biofouling‐promoting bacteria. Our findings reveal a previously unrecognised connection between anthropogenic bacterial contamination and biofouling dynamics, establishing bacteriophages as an environmentally sustainable strategy for controlling biofilm‐mediated larval settlement in marine industries.

## Graphical abstract

Anthropogenic‐derived *E. cloacae* colonises marine cooling water systems, where chlorine exposure drives cross‐resistance and enhances biofilm formation, creating hotspots for barnacle larval settlement. This work reveals a previously unrecognised mechanism linking microbial pollution to marine biofouling. A native bacteriophage isolated from the same system effectively disrupts these resistant biofilms and reduces larval settlement, offering a sustainable biofouling control strategy.

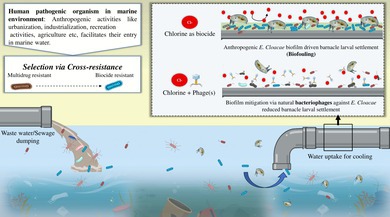

## 1 Introduction

Anthropogenic activities are increasingly introducing human pathogens into marine ecosystems through industrial discharge and wastewater effluent (An et al. 2020; Rangel‐Buitrago et al. 2024). These pathogenic microbial introductions represent an emerging environmental hazard that can disrupt marine ecosystem dynamics and potentially impact human health via direct exposure during swimming, fishing, bathing, or indirectly through seafood contamination (Asif et al. 2024; Shuval 2003). While several studies have examined the persistence and proliferation of these organisms in marine environments (Asif et al. 2024; Jurelevicius et al. 2021; Li et al. 2024), limited information is available on how man‐made marine systems like cooling water circuits, desalination plants, ports, or other coastal discharges influence their survival (Qiao et al. 2025; Sharma et al. 2025). This knowledge gap is critical given that such facilities routinely employ biocides to control biofilms and biofouling (Satasiya et al. 2025), while emerging evidence suggests a strong association between biocide resistance and antibiotic resistance (Hou et al. 2019; Khoo et al. 2022; Xiao et al. 2021). Consequently, one may presume that these systems may serve as selective environments that promote the growth and propagation of biocide and multidrug‐resistant pathogens (cross‐resistance), posing significant public health concerns.

Beyond public health concerns, the colonisation of surfaces by biocide‐resistant pathogenic microorganisms in the form of biofilms can substantially compromise the operational and structural integrity of cooling‐water systems. Notably, such biofilms may influence the settlement of invertebrate larvae and subsequent macrofouling, one of the major challenges in maintaining efficient industrial cooling systems. Microbial biofilms are well documented to play a pivotal role in inducing invertebrate larval settlement, particularly in barnacle larvae (Dobretsov and Rittschof 2020; Peng, Liang, Xu, et al. 2020; Siddik and Satheesh 2019; Tait and Havenhand 2013). However, the role of biocide‐resistant, non‐native pathogenic microbial biofilms in promoting barnacle larval settlement, and thereby contributing to overall biofouling, remains largely unexplored. Moreover, there are limited studies on alternative strategies to manage such resistant biofilms, particularly in systems where chemical biocides remain the only practical and economically viable control option.

The use of conventional biocides such as chlorine presents two major challenges. First, their broad‐spectrum activity means that excessive application leads to substantial biodiversity loss. Chemical pollutants, including biocides, have been identified as the third leading driver of global biodiversity decline (Sigmund et al. 2023). Second, biocides demonstrate significantly reduced efficacy against biofilms compared to planktonic cells, primarily due to diffusion and penetration barriers created by extracellular polymeric substances (EPS) secreted within biofilms. Notably, biofilm‐resident cells can experience up to 1000‐fold lower biocide concentration than their planktonic counterparts (Bridier et al. 2011). These limitations, coupled with the increasing prevalence of biocide resistance, necessitate the development of alternative strategies for managing industrial biofilms.

Bacteriophages, viruses that specifically infect bacterial hosts, represent a promising biological approach for controlling bacterial populations across diverse environments (Harshitha et al. 2022). Their high host specificity, self‐replicating capacity (burst sizes of 50–200 progeny for most lytic phages), and natural origin make them attractive, environmentally sustainable alternatives to conventional antimicrobial agents (Xue et al. 2024). While bacteriophage applications have been extensively documented in medical, food safety, poultry, and aquaculture sectors (Khan and Joshi 2024), their potential for industrial biofilm control remains underexplored.

In this study, we provide the first evidence linking the human opportunistic pathogen *Enterobacter cloacae* , isolated from a marine cooling‐water circuit, to the promotion of barnacle larval settlement. Our findings demonstrate that *E. cloacae* biofilms function as potent settlement cues for barnacle larvae, establishing a previously unrecognised connection between terrestrial bacteria and marine biofouling processes. Furthermore, we provide proof of concept for the targeted elimination of *E. cloacae* from biofilms using bacteriophages as an efficient biofouling control strategy. This bacteriophage‐mediated approach effectively reduces larval settlement while offering an environmentally sustainable alternative to conventional chemical biocides, which are limited by broad‐spectrum toxicity, biocide resistance, and environmental persistence.

## 2 Materials and Methods

### 2.1 Rearing of *Chaetoceros Calcitrans* as a Feed for Barnacle Larvae

F/2 media (f/2 Guillards Marine Enriched Solution (10X), Hi‐media) was prepared and autoclaved in fresh and 0.45 μm filtered seawater. The mid‐log phase culture of *Chaetoceros* was inoculated into the fresh media as a 5% inoculum. The suspension was maintained at 25°C and 110 rpm under a 12‐h light cycle (400 μmol photons m^−2^ s^−1^). L‐D cycles for growth. The colour of the growing culture was monitored daily until it reached dark brown.

### 2.2 Rearing of Barnacle Larvae for the Settlement Assay (Figure 1B)

**FIGURE 1:**
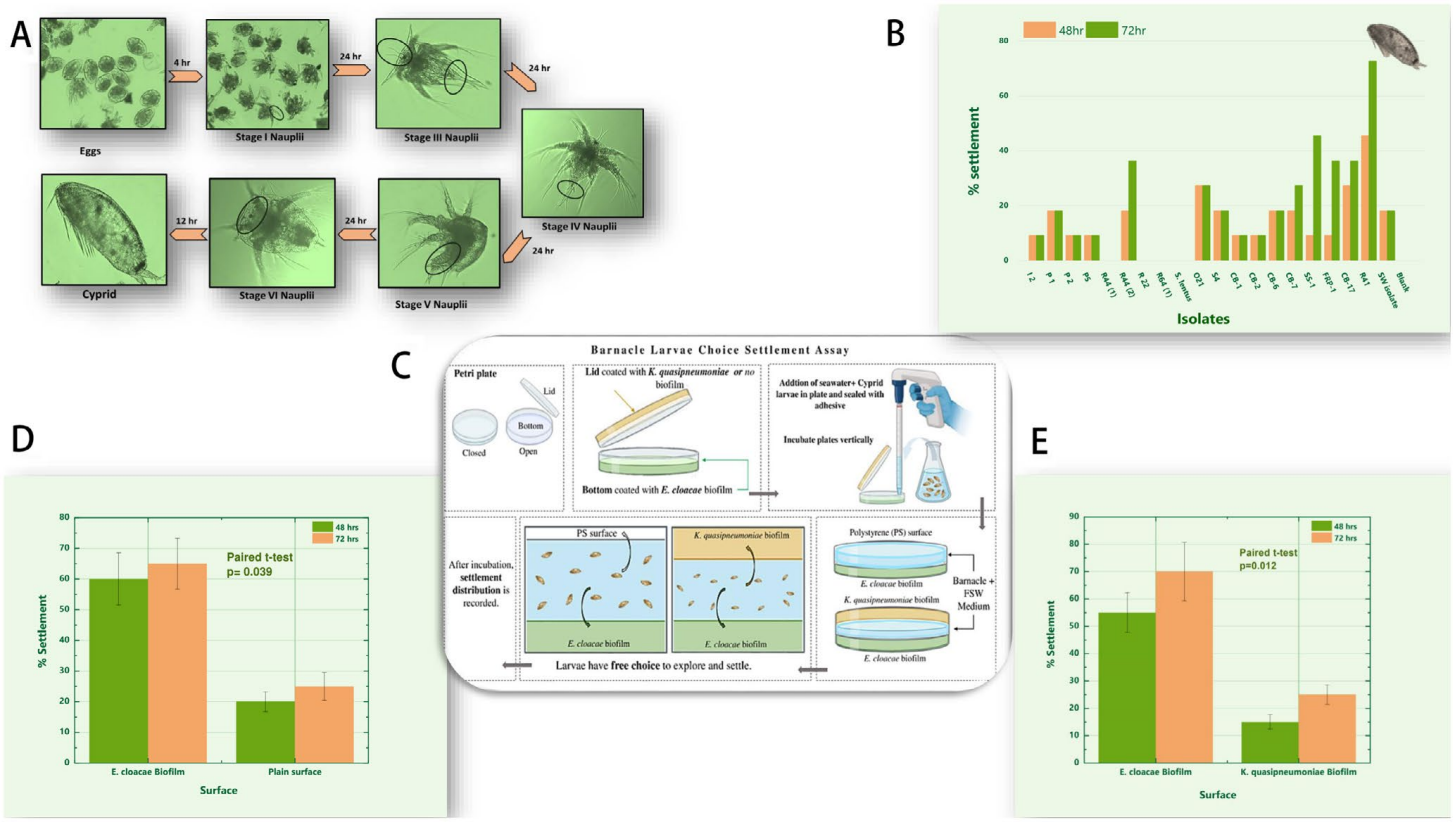
(A) Developmental stages of barnacle larval metamorphosis from egg to cypris (competent settling stage) under laboratory conditions. (B) Representative data on the isolation of larval settlement‐inducing, biofilm‐forming bacteria from the marine cooling water system. (C) Schematic representation of the larval settlement choice assay experimental setup. (D) Percentage settlement of cypris larvae on *E. cloacae* biofilms in choice assays comparing settlement preferences between *E. cloacae* biofilm‐coated surfaces, plain control surfaces, and (E) *K. quasipneumoniae* biofilm‐coated surfaces.

Adult barnacles with mature brooding pouches are collected from the fouled surface, cleaned, and exposed to heat stress under shady sunlight. Afterward, these barnacles were placed in filtered seawater, prompting them to release eggs and stage I Nauplii larvae. The Nauplii larvae were separated from eggs and other debris by exposing them to a light source from the inside of a covered beaker (phototactic movement). Subsequently, the larvae were transferred to a covered beaker at a concentration of 1 larva/mL in 300 mL of seawater. Immediately after transfer, the larvae metamorphose to stage II and are ready to feed. The larvae were fed with previously grown 2.5 × 10^6^ cells/mL of *Chaetoceros*. Daily cleaning and feeding cycles were repeated till they reached the Cypris stage after 5–6 days. Over 5–6 days, the larvae metamorphose into cypris, which are identified by their distinct shape and movement. The cypris were collected in filtered seawater and stored at 4°C for 3 (three) days to enhance their competence for settlement (Maréchal et al. 2012). After three days, the settlement assay was performed under various conditions. The % settlement was calculated by visually counting the number of settled larvae (those that followed settlement with metamorphosis into juveniles) compared to the total number of larvae present. To avoid counting loosely bound larvae, the plates were shaken extensively, and larvae that had metamorphosed to juveniles only were counted. For each condition, three replicates of 6 (six) larvae each (six larvae/well) were used to generate statistically validated data.

### 2.3 Isolation of Larval Settlement‐Inducing Bacteria From the Cooling Water System

For the screening of larval settlement‐inducing marine bacterial isolates, an overnight‐grown culture of previously isolated bacteria was inoculated in 6‐well plates in Zobel Marine Broth and incubated for 24 h at 30°C. The next day, the supernatant was decanted, and each well was washed with sterile seawater to remove loosely bound cells. After washing, each well was inoculated with previously grown 6 (six) Cypris larvae along with 3 mL of sterile seawater. The plates were incubated at 23°C, covered with aluminium foil under stagnant conditions (Low light and stagnant conditions were maintained to promote maximum larval settlement at the bottom of the wells and to minimise settlement on the walls, which can occur due to the phototactic migration of larvae). Cypris settlement was assessed after 24, 48, and 72 h.

### 2.4 Settlement Choice Assay for Reconfirming Biofilm‐Induced Settlement (Figure 1C)

To generate supporting evidence for biofilm‐induced larval settlement, we conducted a two‐choice settlement assay in which cypris larvae were provided with two surfaces to assess their settlement preferences. Two experimental conditions were tested: (1) larvae were offered a surface conditioned with an *Enterobacter cloacae* biofilm alongside a clean, unconditioned surface, enabling comparison between a biological cue and an abiotic control; and (2) a direct comparison between two biofilm‐conditioned surfaces, (A) *E. cloacae* biofilm and (B) *Klebsiella quasipneumoniae* biofilm.

For each assay, biofilms were developed on the bottom halves of sterile 30 mm Petri plates {30 mm diameter and 22 mm height (11 mm × 2)} by inoculating the target bacterial strain into sterile media and incubating for 24 h at 30°C. Following incubation, the supernatant was removed, and the surfaces were gently washed twice with sterile seawater. The biofilm‐bearing halves of two Petri plates were then joined together from their rim and sealed with adhesive (Araldite) to create a chamber. Six cypris larvae were randomly suspended in filtered seawater and introduced into the space between the sealed plates via a small hole created on the side, which was subsequently sealed. The plates were incubated in a vertical orientation to facilitate observation of larval settlement.

In the single‐biofilm setup, one side of the chamber contained a biofilm, while the other side remained unconditioned. Settlement percentages were determined by counting the number of larvae attached to either surface at 48 and 72 h post‐introduction and metamorphosing into juveniles.

### 2.5 Investigation on the Role of Cell‐Bound or Diffusible Molecule(s) on the Induction of Larval Settlement

To evaluate the influence of specific cellular components on larval settlement behaviour, fractionated components derived from *Enterobacter cloacae* biofilms and planktonic cultures were tested. Biofilms were established in sterile 12‐well polystyrene plates and subsequently disrupted via sonication on ice to achieve complete cell lysis. The resulting lysates were centrifuged at 8000 rpm for 10 min to obtain distinct pellet and supernatant fractions. Planktonic cultures were subjected to identical lysis and fractionation procedures to ensure experimental consistency and enable direct comparison between biofilm and planktonic‐derived components.

Settlement assays were conducted using intact biofilms, intact planktonic cells, and sterile seawater as experimental controls. Each test fraction was transferred to sterile 6‐well plates containing 3 mL of filtered seawater and cypris larvae in triplicate. Covered plates were incubated for 72 h at 23°C. Larval settlement rates were quantified at 48‐ and 72‐h time points, and the percentage of successfully settled larvae was calculated for statistical analysis.

### 2.6 Whole Genome Sequencing of Larval Settlement‐Inducing *E. cloacae*

The whole genome sequence of larval settlement‐inducing *E. cloacae* was performed using the Nanopore sequencing platform. Total genomic DNA was isolated using the DNeasy PowerLyzer Microbial Kit (Qiagen) from 1.8 mL of an overnight‐grown culture. The DNA was quantified using a Qubit fluorometer. 1 μg of DNA was taken for library preparation using the ligation sequencing kit (Oxford Nanopore) as per the protocol provided by the manufacturer. Briefly, the DNA was subjected to end repair using the NEBNext Ultra II kit, followed by barcode ligation and adapter ligation. The final library was placed on a MinION flow cell for sequencing. Post sequencing, the raw sequence was obtained, and a comprehensive analysis was performed on the BV‐BRC web portal (https://www.bv‐brc.org). Alternatively, various bioinformatics analyses were performed using various tools available on the Galaxy portal (https://usegalaxy.org).

### 2.7 Isolation and Characterisation of *E. cloacae* ‐Specific Bacteriophages From the Cooling Water Circuit

For the isolation of *E. cloacae* ‐specific bacteriophages, the same seawater sample from which *E. cloacae* was originally isolated was utilised. The filtered seawater (filtered using a 0.22 μm filter) was mixed with 2X Zobel Marine Broth (1:1 v/v) and inoculated with an overnight culture of *E. cloacae* . The mixture was incubated at 30°C for 24 h, followed by storage at 4°C for 48 h to enrich for bacteriophages. Following enrichment, the mixture was centrifuged, and the supernatant was filtered through a 0.22 μm membrane filter to obtain the amplified phage lysate. The presence of *E. cloacae‐*specific phages was confirmed by spot‐testing the lysate on a bacterial lawn of *E. cloacae* .

The phages were subsequently purified using standard plaque assay techniques. Based on distinct plaque morphologies, five bacteriophages were isolated and propagated through multiple rounds of amplification on the host strain. Restriction Fragment Length Polymorphism (RFLP) analysis was performed to confirm that the isolated phages were genetically distinct from one another.

To evaluate the growth inhibition efficiency of the isolated phages, growth inhibition assays were conducted by co‐inoculating *E. cloacae* cells with individual phages and phage cocktails at varying multiplicities of infection (MOI) of 1, 10, and 100 (i.e., 10^6^,10^7^ and 10^8^ PFU/mL respectively) in 96‐well microtiter plates inoculated with 10^6^ CFU/mL of overnight grown culture of *E. cloacae* . Cultures were incubated at 30°C with intermittent shaking using a multimode plate reader (Tecan Life Sciences). Growth kinetics were monitored by measuring optical density at 600 nm (OD_600_) at hourly intervals. Six replicates of each combination were used to get statistically validated data. For quantification of biofilm inhibition efficiency, a crystal violet (CV)‐based biofilm assay was performed following previously established protocols (Khan and Joshi 2024; Niu and Gilbert 2004). To assess the biofilm removal capability of the isolated bacteriophages, 24‐h‐old biofilms grown in 96‐well plates were treated with individual phages and phage cocktails at concentrations of 10^12^, 10^11^, and 10^10^ PFU/mL. After 6 h of incubation, residual biofilm biomass was quantified using the CV assay following standard washing procedures.

### 2.8 Bacteriophage‐Mediated Larval Settlement Inhibition

To demonstrate the proof of concept for bacteriophage application in biofouling control, larval settlement experiments were conducted following the protocol described in Section 2.2. *E. cloacae* biofilms were treated with a cocktail of bacteriophages, and larval settlement responses were evaluated. Untreated *E. cloacae* biofilm served as the positive control, while sterile seawater was used as the negative control.

Additional controls were included to establish that the observed settlement responses were mediated by biofilm and not by bacteriophages alone. These controls comprised: (i) heat‐inactivated phages, and (ii) bacteriophages in seawater without bacterial biofilm.

To further validate that phage‐mediated settlement inhibition was governed through biofilm disruption, two experimental conditions were tested: (i) phages co‐inoculated with *E. cloacae* from time zero (T_0_) to prevent biofilm formation, and (ii) phages added to pre‐established biofilms to remove biofilm.

### 2.9 Targeted Elimination of *E. cloacae* Biofilm for Larval Settlement Inhibition

To demonstrate the environmental sustainability of bacteriophages over conventional biocides, experiments were performed to assess the possibility of reducing larval settlement by targeted elimination of the organism of interest (here larval settlement‐inducing *E. cloacae* ). A dual‐species biofilm comprising *Enterobacter cloacae* and *Klebsiella quasipneumoniae* was developed and independently treated with bacteriophages specific to *E. cloacae* and *K. quasipneumoniae* before performing the larval settlement assay. % Settlement was determined under each experimental condition. Unrested single‐species biofilm was used as a positive control, and seawater alone was taken as a negative control. All the experiments were carried out in 3 replicates.

## 3 Results

### 3.1 Isolation of Larval Settlement‐Inducing Bacteria From the Cooling Water System

Based on established literature documenting the role of bacterial biofilms in barnacle larval settlement (Bacchetti De Gregoris et al. 2012; Dobretsov and Rittschof 2020; Khandeparker et al. 2006; Li et al. 2010; Rajitha et al. 2024; Thiyagarajan (Rajan) et al. 1999) and extensive biofouling by barnacles (*Amphibalanus reticulatus*) reported in a coastal power plant situated on the Bay of Bengal coast (Rajitha et al. 2024), we attempted to isolate bacteria responsible for inducing larval settlement from the cooling water system of an operational power plant. Amongst 150 isolates screened, one isolate induced larval settlement by > 70% compared to the control (Figure 1B). To further validate the settlement‐inducing properties of this isolate, we conducted settlement choice assays in which barnacle larvae were presented with two alternative surfaces for settlement selection (Figure 1C). As shown in Figure 1D,E, there was a significantly higher settlement preference of barnacle larvae on surfaces colonised with the selected isolate compared to the control surface, thereby reconfirming its larval settlement‐inducing properties.

### 3.2 Identification of Cellular Component(s) Involved in Settlement Induction

**FIGURE 2:**
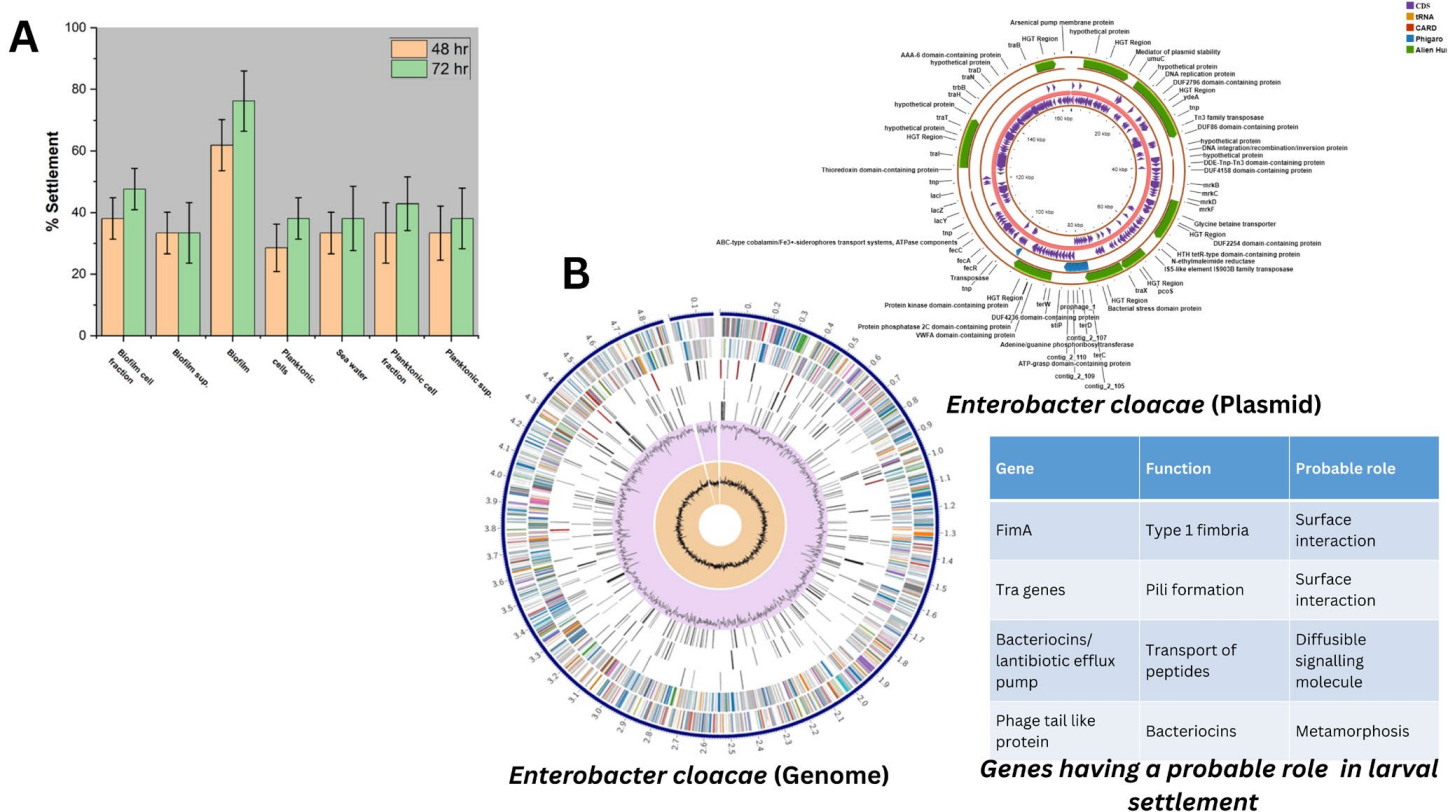
(A) Effect of different cellular components on cypris larval settlement behaviour. (B) Circular genome map, plasmid organisation, and putative genes potentially involved in barnacle larval interactions.

Two primary mechanisms may mediate microbial‐induced larval settlement: (1) signalling through soluble extracellular molecules, or (2) signalling through cell‐associated molecules such as surface receptors or membrane‐bound compounds. To elucidate the probable mechanism underlying larval settlement induction, we collected and tested various fractions, including biofilm components and planktonic cells. Results demonstrated significantly higher settlement rates when the cellular fraction of biofilms was employed compared to supernatant or planktonic cell treatments (Figure 2A). This observation clearly indicates the pivotal role of biofilm and cell‐bound components in mediating larval settlement behaviour, suggesting that surface‐associated rather than soluble factors serve as the primary settlement cues. However, further experimentation is required to identify the cell‐bound molecule(s) involved in settlement induction.

### 3.3 Whole Genome Sequencing, Identification, and Comparative Assessment With Reference Genome

With strong evidence indicating the larval settlement‐inducing properties of the isolate, we conducted whole‐genome sequencing to identify the organism and determine the presence of potential genes responsible for these settlement‐inducing properties. Based on comparative 16S rRNA gene sequence and Multi‐Locus Sequence Typing (MLST), the organism was identified as *Enterobacter cloacae* , sequence type rST‐224,631. The genome sequence was submitted to the NCBI database with accession numbers CP183059.1 (genome) and CP183058.1 (plasmid). We designated this isolate as *Enterobacter cloacae BARC_01*. The circular genome and plasmid are shown in Figure 2B along with key genomic features that may be involved in the induction of larval settlement. The sequence revealed the presence of genes associated with cell appendages, such as fimbria, pili, a bacteriocin/lantibiotic efflux ABC transporter, and a phage tail fibre‐like protein, which were previously reported to contribute to invertebrate larval settlement (Liang et al. 2020; Shikuma et al. 2014). Additionally, the genome was found to contain genes associated with antibiotic and biocide resistance. As indicated in Table S1, the genome consists of katG, CMH family, MarA, MarB, and MarR along with genes associated with efflux pumps like AcrAB‐TolC, EmrD, TolC/OpmH, etc., which were previously reported to contribute towards robust antibiotics and biocide resistance in bacteria (Dulanto Chiang and Dekker 2024; Hajiagha and Kafil 2023; Sharma et al. 2019; Wand and Sutton 2022). In addition, as shown in Figure S1, there were 9 sequences similar to pro‐phages with a maximum score of 0.873 found using the PhageBoost program (https://phageboost.ku.dk/).

**FIGURE 3:**
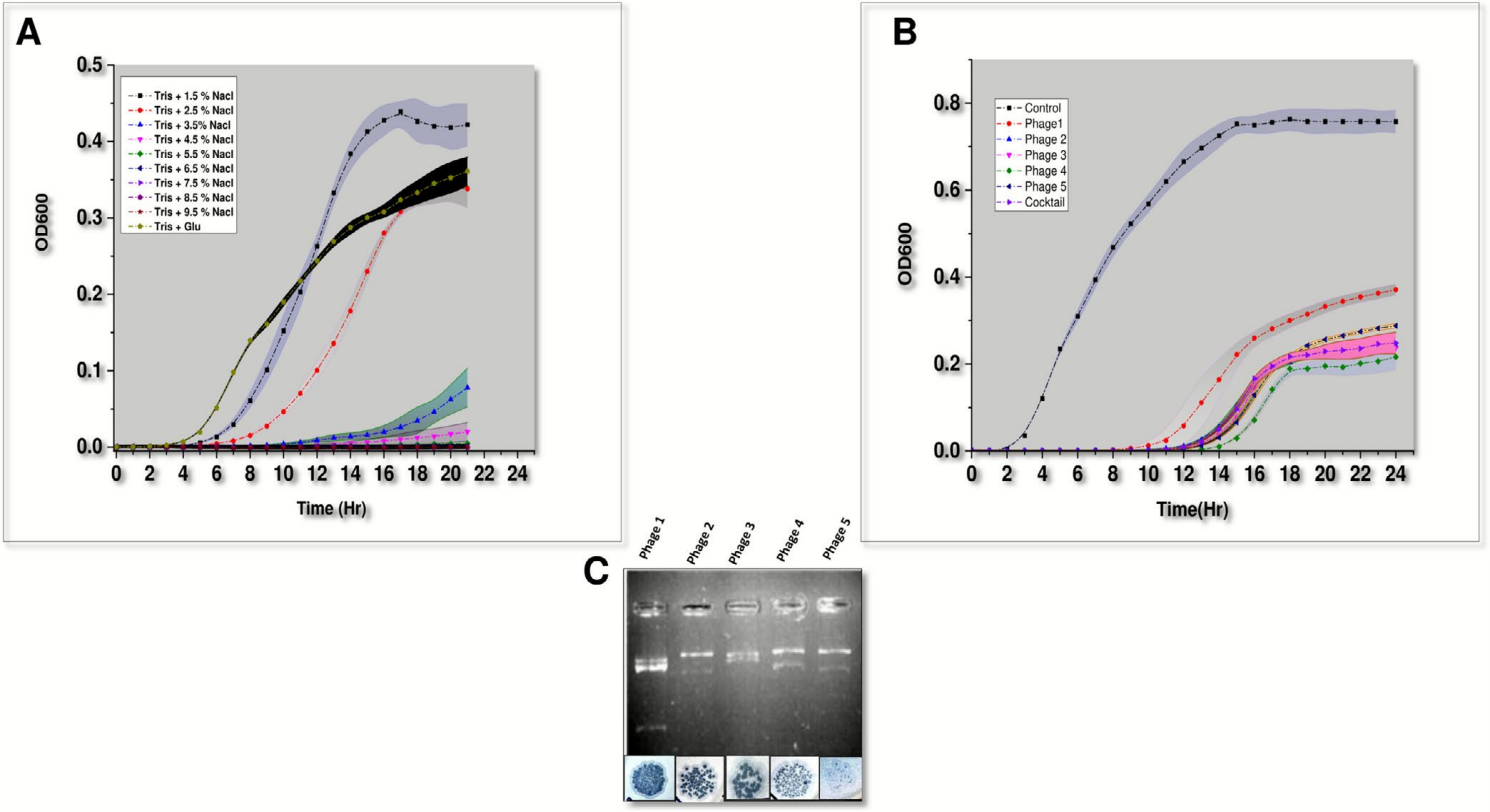
(A) Effect of salt concentration on *E. cloacae* growth kinetics. (B) Planktonic growth inhibition of *E. cloacae* by specific bacteriophages. (C) Restriction Fragment Length Polymorphism (RFLP) profile of isolated bacteriophages.

Interestingly, a detailed genome comparison with the *E. cloacae* ATCC 13047 reference genome (accession number: NC_014121.1) revealed a very high degree of similarity, with 4304 core loci (84.8%) present at 100% prevalence. The Average Amino acid Identity (AAI) with the reference genome was found to be 99.3% (kindly see Figure S2 for Scatterplot and AAI histograms), and Average Nucleotide Identity (ANI) was found to be 98.48%. Additionally, based on rMLST profile matching, the isolate was found to belong to a sequence type (rST‐224631) originally isolated from clinical samples, raising the possibility that our isolate may not be a marine strain but rather one that entered through anthropogenic activity. To verify its origin, we investigated the influence of salt concentration on the growth of *E. cloacae BARC_01*. As shown in Figure 3A, significantly higher growth (two‐sample *t*‐test: *p* < 0.05, without salt vs. different concentrations of salt) was observed in media without the addition of salt compared to higher salt concentrations, further supporting the non‐marine origin of *E. cloacae BARC_01*.

### 3.4 Isolation and Characterisation of *E. cloacae* ‐Specific Bacteriophages From the Cooling Water Circuit

Biofouling represents a significant operational challenge for industries utilising marine water systems. The accumulation of marine invertebrates (biofouling) in seawater transport pipelines results in reduced flow capacity, elevated pumping energy requirements, and accelerated material degradation. Consequently, most operational facilities employ biocide treatments to control biofouling. While effective, conventional biocides present substantial environmental concerns, including adverse effects on marine biodiversity and direct and indirect toxicity to non‐target organisms, including humans. Given these limitations of traditional biocidal approaches and compelling evidence demonstrating the larval settlement‐inducing properties of specific bacterial biofilm, the targeted elimination of this specific bacterial strain emerges as a promising strategy for reducing larval settlement and subsequent biofouling. Bacteriophages are emerging as a promising alternative to various biocides, primarily due to their host specificity and active biofilm penetration capabilities. Consequently, we have attempted to isolate bacteriophages specific to *E. cloacae BARC_01* (Host range: Table S2) from the cooling water from where it was previously isolated. As shown in Figure 3B, five bacteriophages having distinct plaque morphology (plaque diameter, clarity, edge, etc.) were isolated and purified. Furthermore, RFLP was performed to verify the distinct identity of isolated bacteriophages (Figure 3C).

**FIGURE 4:**
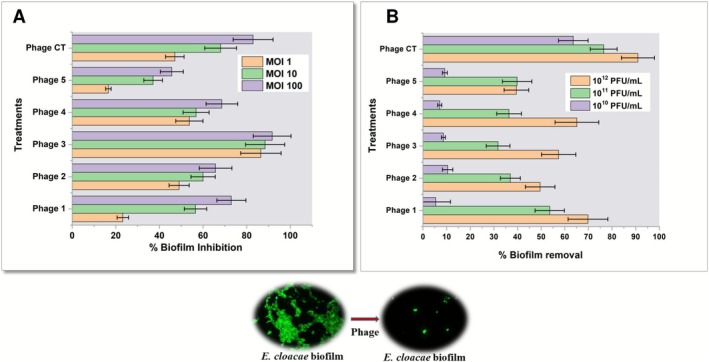
(A) Inhibition of biofilm formation by *E. cloacae* ‐specific bacteriophages at different Multiplicities of Infection (MOI) values. (B) Removal of preformed biofilm by *E. cloacae* ‐specific bacteriophages.

After isolating those phages, we evaluated their growth and biofilm inhibition efficacy. As shown in Figure 3B, all the isolated bacteriophages were found to significantly inhibit the planktonic bacterial growth (two‐sample *t*‐test; *p* < 0.05, control vs. phage‐treated). Furthermore, the phage cocktail reduced biofilm formation by over 82% at an MOI of 100 compared to the untreated control (Figure 4A). Additionally, they were also able to eradicate more than 90% of preformed biofilms (Figure 4B). The strong antibiofilm activity of the isolated phages suggests their potential to eliminate biofilm formation by *E. cloacae* BARC_01, thereby reducing larval settlement; hence, they were tested.

### 3.5 Bacteriophage‐Mediated Larval Settlement Inhibition

**FIGURE 5:**
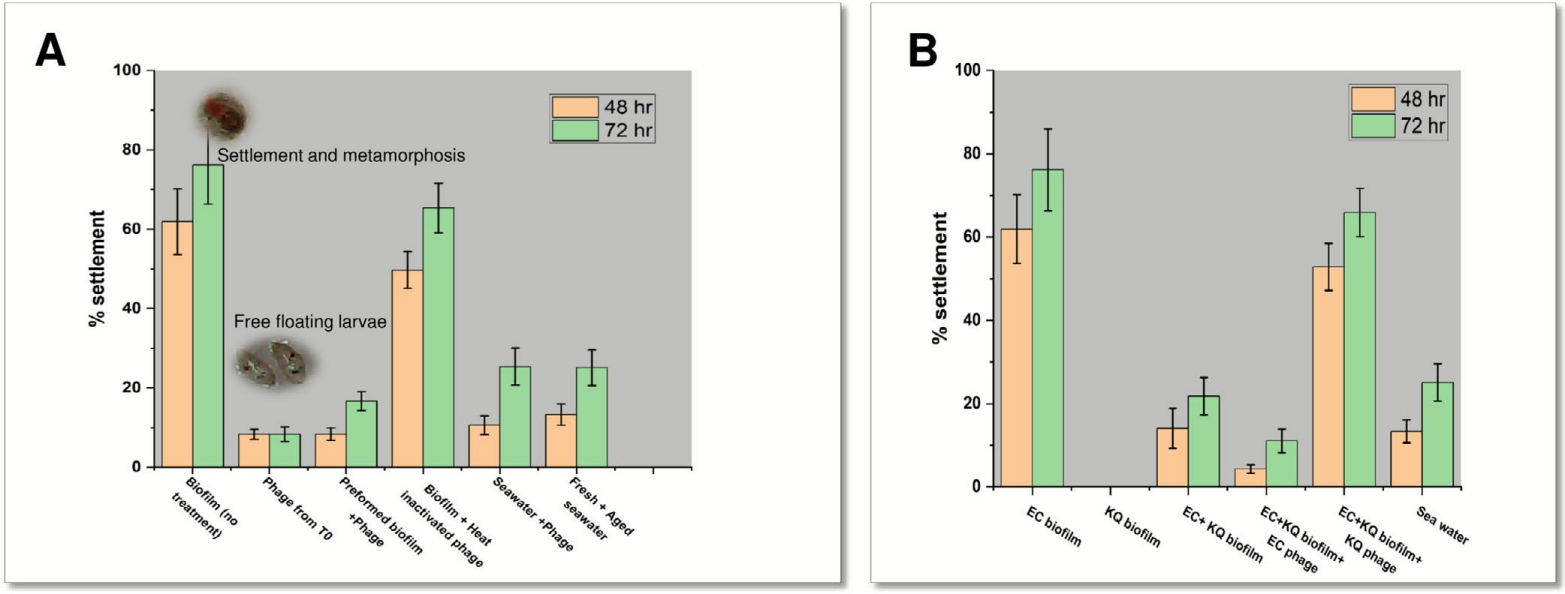
(A) Effect of bacteriophage treatment on settlement of barnacle larvae *on E. cloacae* biofilm. (B) Proof of concept demonstration of selective elimination of settlement‐inducing bacteria from dual‐species biofilms as an effective strategy for inhibiting barnacle larval settlement and biofouling control.

After establishing the larval induction capabilities of *E. cloacae BARC_01* and isolating effective bacteriophages specific to *E. cloacae BARC_01*, we have evaluated the possibility of using bacteriophages to eliminate *E. cloacae BARC_01* biofilm and thereby reduce larval settlement. As shown in Figure 5A, there was a significant reduction in larval settlement when bacteriophages were used to inhibit the biofilm formation by *E. cloacae BARC_01*. In addition, an almost identical reduction in settlement was observed when the preformed biofilm of *E. cloacae BARC_01* was treated with bacteriophage prior to the settlement assay. However, there was no reduction in settlement when heat‐inactivated bacteriophages were used. These observations reconfirm the larval settlement‐inducing property of the biofilm of *E. cloacae BARC_01* and provide the first direct evidence towards the applicability of bacteriophages to substantially reduce larval settlement or biofouling.

### 3.6 Targeted Elimination of *E. cloacae* Biofilm for Larval Settlement Inhibition

To further validate the anti‐biofouling activity of bacteriophages and assess the feasibility of selectively eliminating specific organisms from a multispecies biofilm using bacteriophages as an environmentally sustainable strategy, we established dual‐species biofilms comprising *Enterobacter cloacae BARC_01* (a settlement‐inducing strain) and *Klebsiella quasipneumoniae* (a strain exhibiting settlement‐inhibitory effects). As shown in Figure 5B, the targeted elimination of *E. cloacae BARC_01* using specific bacteriophages resulted in significant inhibition of larval settlement. Conversely, the selective removal of *K. quasipneumoniae* from the biofilm using its corresponding phages, without impacting *E. cloacae BARC_01*, restored larval settlement to over 70%. These results offer two key insights: (1) they reinforce the role of *E. cloacae BARC_01* in promoting cypris settlement, and (2) they provide proof‐of‐concept for phage‐mediated biofouling control through the targeted removal of organisms from biofilms that influence larval settlement.

## 4 Discussion

Our study suggests a potential linkage between anthropogenic microbial contaminants and biofouling by identifying *Enterobacter cloacae* , an opportunistic pathogen of terrestrial origin, as a potent inducer of barnacle (*Amphibalanus reticulatus*) larval settlement in industrial cooling water systems. This finding highlights a previously unrecognised ecological consequence of anthropogenic microbial contamination on enhanced biofouling through the enrichment of non‐native, biofilm‐forming pathogens. We reported that *E. cloacae BARC_01* biofilm enhanced larval settlement, similar to previously reported observations with marine *Pseudoalteromonas* and *Vibrio* spp. (Freckelton et al. 2022, 2017; Liu et al. 2024; Peng, Liang, Xu, et al. 2020; Tait and Havenhand 2013).

Antimicrobial resistance (AMR) is one of the major global challenges, and therefore, significant efforts have been directed towards understanding the spread of AMR across various environmental settings. Many reports clearly establish the presence of AMR genes and organisms in the marine environment (Bonanno Ferraro et al. 2024; Jurelevicius et al. 2021). Interestingly, there was significant genomic and physiological similarity between pathogens isolated from the marine environment and their terrestrial counterparts, indicating they spread through anthropogenic activities (Parab et al. 2025; Pérez‐Etayo et al. 2020). Higher growth rates at lower salt concentrations, identical rMLST profiles, over 95% AAI and ANI similarity with the *E. cloacae* reference strain, and a high degree of genetic concordance across shared loci collectively indicate a terrestrial, pathogenic, and non‐marine origin of *E. cloacae BARC_01*. Unlike specific genes that confer resistance against individual antibiotics, efflux pumps provide broad‐spectrum resistance against diverse antimicrobials, including antibiotics, biocides, heavy metals, and other toxic compounds (Nishino et al. 2021; Qiu et al. 2023; Wand and Sutton 2022). This efflux pump‐mediated cross‐resistance enables biocide‐resistant organisms to survive in the presence of antibiotics and vice versa. The presence of multiple genes associated with efflux pumps (i.e., AcrAB‐TolC, AcrAD‐TolC, AcrEF‐TolC, AcrZ, EmrAB‐TolC, EmrD, etc.) in the genome of *E. cloacae BARC_01* suggests that efflux pumps‐mediated cross‐resistance mechanisms may facilitate the survival and proliferation of *E. cloacae BARC_01* in a chlorine‐dosed cooling water system.

The unwanted accumulation of biological material or biofouling on the components of the cooling water system or any other marine structure is considered to be a major operational challenge (Satasiya et al. 2025). It has been extensively reported that bacterial biofilms play a significant role in the recruitment of larvae of invertebrates and thereby facilitate the settlement and macrofouling (Dobretsov and Rittschof 2020; Freckelton et al. 2022; Hadfield 2011). There are three broad mechanisms by which bacteria interact with invertebrate larvae: (1) Secretion of water‐soluble molecules like quorum‐sensing molecules, peptides, etc. (Tait and Havenhand 2013), (2) Secretion of larger biomolecules like protein, DNA, or enzymes (Shikuma et al. 2014), and (3) interaction via surface‐bound molecules like fibri, flagella, receptors, and exopolymeric substances (Freckelton et al. 2022; Peng, Liang, Chang, et al. 2020). Our experiments suggest that only the intact biofilm and cellular fraction of *E. cloacae BARC_01* trigger larval settlement. In contrast, planktonic cells and their fractions showed no effect, pointing to a likely involvement of biofilm‐associated, cell‐bound molecules in inducing the larval settlement. Additionally, the presence of genes associated with the synthesis of phage tail fibres like protein, fimbria, pili, adhesins (plasmid coded), and efflux pumps (genome coded) (Figure 2B), which have been linked to both strong biofilm formation and larval settlement in other organisms, further supports the role of biofilm‐associated, cell‐bound molecules in the induction of larval settlement (Dobretsov and Rittschof 2020; Freckelton et al. 2022; Kitade et al. 2024; Liu et al. 2024). These findings suggest that anthropogenic strains may be using the conserved signalling pathways to manipulate larval behaviour or induce settlement and metamorphosis. However, more research is needed to identify the specific molecular triggers or signals responsible for the induction of larval settlement.

Most of the marine Industries currently rely on broad‐spectrum biocides, including chlorine, chlorine dioxide, quaternary ammonium compounds, and various physicochemical methodologies for mitigating biofouling (Satpathy et al. 2010). However, these conventional approaches present substantial limitations and environmental risks. For example, chlorine, the most widely used biocide, has been extensively documented to have a significant negative impact on environmental and human health. It has been associated with (1) biodiversity loss, (2) direct toxicity to non‐target organisms, (3) indirect toxicity through disinfection byproduct formation, and (4) operational and occupational hazards (Al‐Abri et al. 2019; Khodary et al. 2010; Venkatnarayanan et al. 2017; Vinitha et al. 2010). Furthermore, the rapid rise of biocide‐resistant organisms and associated cross‐resistance to multidrug resistance highlights the critical need for alternative biofouling mitigation strategies that minimise environmental impact while maintaining efficacy. It is becoming clear that only certain bacterial biofilms play a critical role in larval settlement, allowing for more targeted interventions. Selective targeting of larval settlement‐inducing organisms can achieve desired biofouling control while preserving non‐target microorganisms and maintaining ecological balance (Khan et al. 2025).

Bacteriophages are known to have very high specificity towards their host and, therefore, present a promising solution for the targeted elimination of larval settlement‐inducing organisms. Additionally, unlike conventional antimicrobial agents, bacteriophages exhibit unique biofilm penetration capabilities mediated by depolymerase enzymes present on their tail fibre. These depolymerase enzymes facilitate matrix degradation through the hydrolysis of extracellular polymeric substances (EPS), enabling phages to penetrate deep inside the biofilm and eliminate the host (Chang et al. 2022; Ma et al. 2018). Consequently, bacteriophages can be an ideal choice for eliminating larval settlement‐inducing organisms and their biofilms.

Here we have demonstrated that bacteriophages specific to *E. cloacae BARC_01*, isolated from the same water sample, effectively inhibit bacterial growth and biofilm formation, resulting in significant reductions in larval settlement. Importantly, bacteriophages were also able to effectively eliminate preformed biofilm of *E. cloacae BARC_01*, highlighting their potential application in real‐world scenarios where systems might already be colonised by larval settlement‐inducing biofilms. Additionally, the selective removal of *E. cloacae BARC_01* from dual‐species biofilms further supports the applicability of bacteriophages for the targeted elimination of the organism of interest without affecting other organisms.

These findings provide compelling proof‐of‐concept evidence for bacteriophage‐mediated biofouling control. The specificity and biofilm‐penetrating capabilities of bacteriophages offer significant advantages over conventional broad‐spectrum biocides, potentially providing environmentally sustainable biofouling control strategies in marine and industrial applications.

## 5 Conclusion

In conclusion, this study presents the first evidence implicating the anthropogenic, biocide‐resistant, opportunistic pathogen *Enterobacter cloacae* in promoting barnacle larval settlement. Our findings reveal a previously unrecognised link between terrestrial microbial pollution and biofouling, one of the major operational challenges in marine industrial systems. We further demonstrate that biocide dosing practices commonly employed in these industries may paradoxically favour the proliferation of multidrug‐resistant pathogens due to cross‐resistance between biocides and antibiotics.

Although the exact route by which *E. cloacae* entered coastal waters remains unclear, we hypothesise that human activities, particularly the discharge of untreated sewage into marine environments, followed by selective enrichment under chlorinated cooling‐water conditions, facilitated its establishment. To counter the biocide‐resistant, larval settlement‐inducing *E. cloacae* biofilm, we successfully employed naturally occurring bacteriophages isolated from the same chlorinated water samples. These phages selectively inhibited and eliminated *E. cloacae* biofilm in dual‐species biofilms without affecting non‐target organisms, highlighting their effectiveness and host specificity.

This study establishes foundational evidence for the role of anthropogenic pathogens in accelerating biofouling and demonstrates the potential of bacteriophages as sustainable tools for controlling such resistant biofilms. However, further research across multiple sites and system conditions is warranted. Critical challenges, including optimisation of phage dosing, stability, delivery mode, and large‐scale application, must be addressed before bacteriophage‐based strategies can be fully implemented for industrial biofouling control. Additional multiple field trials need to be carried out to establish broader involvement of anthropogenic microbial contaminants in biofouling and validate the scalability and reliability of phage‐mediated mitigation approaches.

## Author Contributions

**Atif Khan:** data curation, formal analysis, investigation, methodology, writing – original draft. **Akash Saha:** software, visualization. **Hiren M. Joshi:** conceptualization, project administration, resources, supervision, writing – original draft, writing – review and editing.

## Funding

The authors have nothing to report.

## Conflicts of Interest

The authors declare no conflicts of interest.

## Supporting information


**Figure S1:** Presence of Pro‐phage sequence in genome of *Enterobacter cloacae BARC_01*.

**Figure S2:** Scattered chart of Average Amino acid Identity (AAI) of *Enterobacter cloacae BARC_01* with reference strain (Accession no: NC_014121.1).

**Table S1:** Presence of antimicrobial resistance genes in the genome of *Enterobacter cloacae BARC_01*.

**Table S2:** Host range determination of *E. cloacae* ‐specific bacteriophages.

## Data Availability

Whole genome sequence of *Enterobacter cloacae BARC_01* was submitted to GenBank with accession numbers CP183059.1 and CP183058.1.
