# Supplementary figures and images for: Anthropogenic Disruption Versus Natural Restoration: Enterobacter cloacae‐Driven Barnacle Larval Settlement and Its Mitigation via Natural Bacteriophages
Source: Microb Biotechnol. 2026 Jan 27;19(1):e70278. doi: 10.1111/1751-7915.70278 (PMC12836379; doi:10.1111/1751-7915.70278)

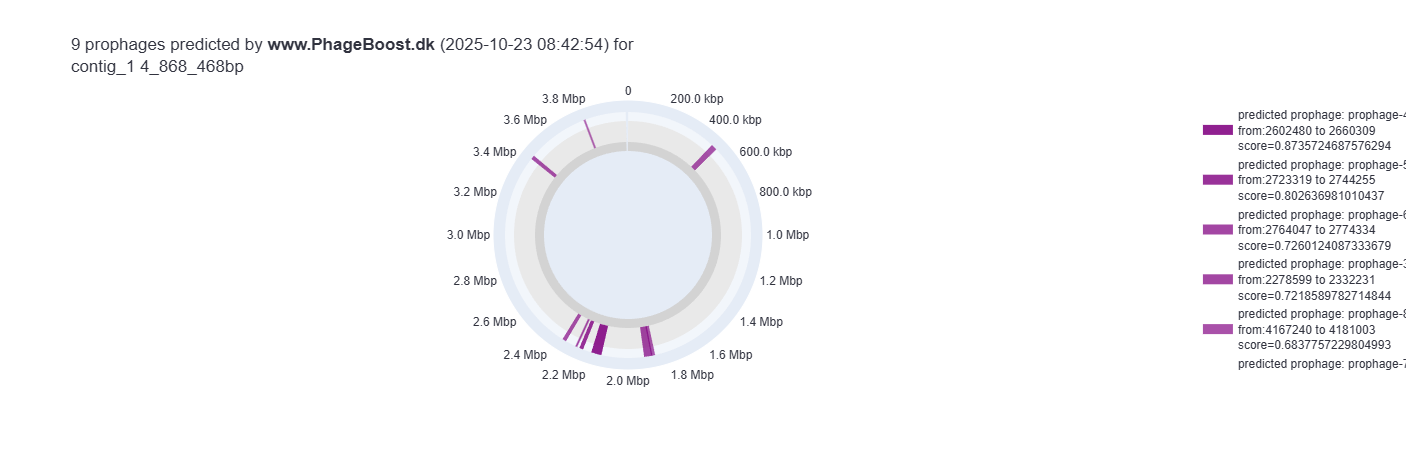

Supplement: Supplementary file 1 — Figure S1: Presence of Pro‐phage sequence in genome of Enterobacter cloacae BARC_01. [file MBT2-19-e70278-s003.tif]

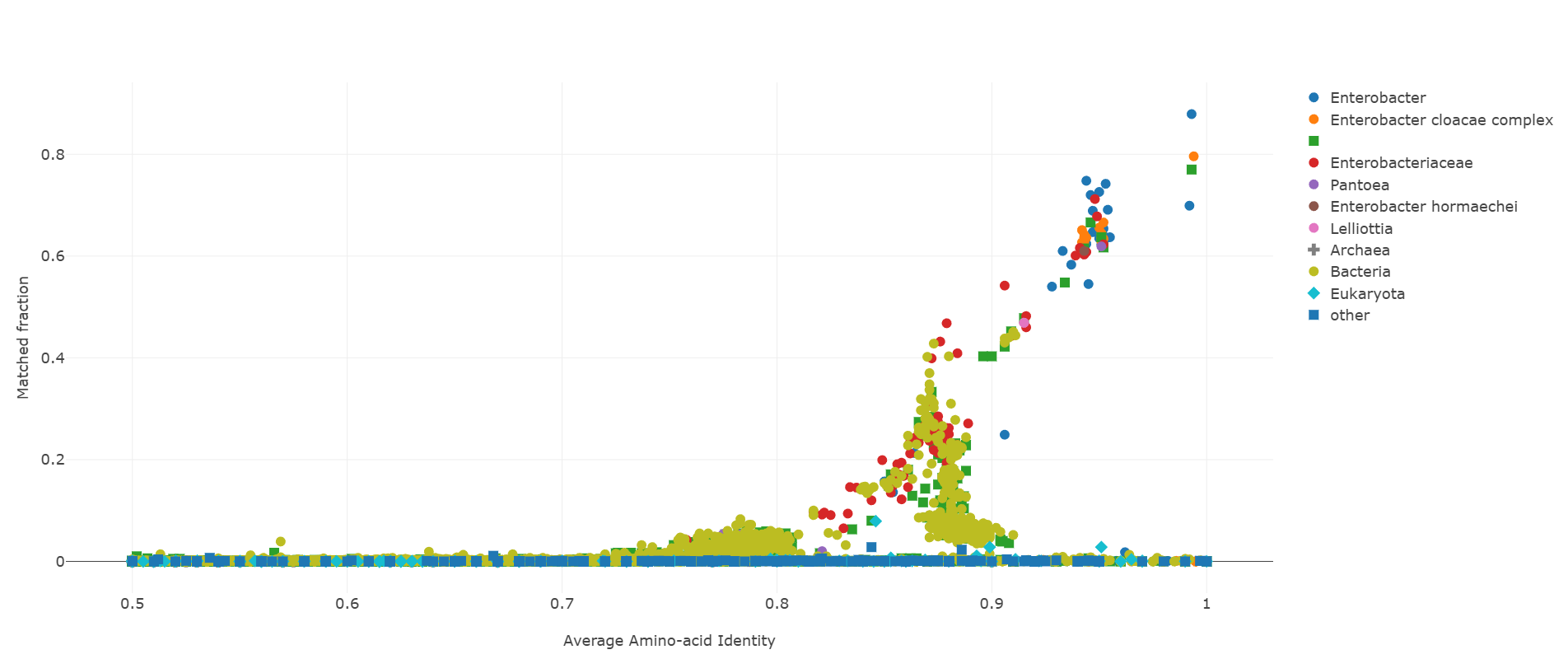

Supplement: Supplementary file 2 — Figure S2: Scattered chart of Average Amino acid Identity (AAI) of Enterobacter cloacae BARC_01 with reference strain (Accession no: NC_014121.1). [file MBT2-19-e70278-s002.tif]
