# Supplementary material for: Anthropogenic Disruption Versus Natural Restoration: Enterobacter cloacae‐Driven Barnacle Larval Settlement and Its Mitigation via Natural Bacteriophages
Source: Microb Biotechnol. 2026 Jan 27;19(1):e70278. doi: 10.1111/1751-7915.70278 (PMC12836379; doi:10.1111/1751-7915.70278)
Supplement: Supplementary file 3 — Table S1: Presence of antimicrobial resistance genes in the genome of Enterobacter cloacae BARC_01. [file MBT2-19-e70278-s001.docx]

Table 1: Presence of antimicrobial resistance genes in the genome of *Enterobacter cloacae BARC_01*

| Antimicrobial Resistance Genes | |
| --- | --- |
| AMR Mechanism | **Genes** |
| Antibiotic activation enzyme | KatG |
| Antibiotic inactivation enzyme | CMH family |
| Antibiotic resistance gene cluster, cassette,or operon | MarA, MarB, MarR |
| Antibiotic target in susceptible species | Alr, Ddl, dxr, EF-G, EF-Tu, folA, Dfr, folP, gyrA, gyrB, inhA, fabI, Iso-tRNA, kasA, MurA, rho, rpoB, rpoC, S10p, S12p |
| Antibiotic target protection protein | BcrC |
| Efflux pump conferring antibiotic resistance | AcrAB-TolC, AcrAD-TolC, AcrEF-TolC , AcrZ, EmrAB-TolC, EmrD, MacA, MacB, MdfA/Cmr, MdtABC-TolC, MdtL, SugE, TolC/OpmH |
| Gene conferring resistance via absence | gidB |
| Protein altering cell wall charge conferring antibiotic resistance | GdpD, PgsA |
| Protein modulating permeability to antibiotic | OccD6/OprQ |
| Regulator modulating expression of antibiotic resistance genes | AcrAB-TolC, EmrAB-TolC, H-NS, OxyR |
