# Supplementary material for: Anthropogenic Disruption Versus Natural Restoration: Enterobacter cloacae‐Driven Barnacle Larval Settlement and Its Mitigation via Natural Bacteriophages
Source: Microb Biotechnol. 2026 Jan 27;19(1):e70278. doi: 10.1111/1751-7915.70278 (PMC12836379; doi:10.1111/1751-7915.70278)
Supplement: Supplementary file 4 — Table S2: Host range determination of E. cloacae ‐specific bacteriophages. [file MBT2-19-e70278-s004.docx]

Table S2: Host range determination of E. cloacae-specific bacteriophages
